# Effect of Time‐Since‐Fire on Ant Communities in a Semi‐Arid Landscape

**DOI:** 10.1002/ece3.73190

**Published:** 2026-03-07

**Authors:** Norma L. Fernando, Nick L. Schultz, Grant Palmer, Philip S. Barton

**Affiliations:** ^1^ Graduate Research School Federation University Australia Mount Helen Victoria Australia; ^2^ Future Regions Research Centre Federation University Australia Mount Helen Victoria Australia; ^3^ School of Life and Environmental Sciences Deakin University Waurn Ponds Victoria Australia

**Keywords:** Anthropocene, biodiversity, Formicidae, insect, mallee landscape, spinifex, succession

## Abstract

Semi‐arid mallee landscapes are shaped by wildfires. Due to climate change, wildfires are expected to become more frequent and intense, making their management a conservation priority. Ants are often used as bioindicators in land management studies, as their composition, richness, and abundance respond to disturbances, including wildfires, both directly and indirectly through habitat modification. In the semi‐arid zone of western New South Wales, Australia, we examined how time‐since‐fire influences ant species richness, abundance, and community composition and assessed whether fire‐induced changes in vegetation composition explain the observed patterns in ant community structure. We sampled ants at five sites that last burned 3, 5, 9, 26, and 34 years ago, respectively. We identified 59 ant species from 20 genera in a total of 16,360 sampled ants. We found that post‐fire ant communities exhibited higher abundance immediately after fire, while species richness increased with time‐since‐fire. Early successional stages with greater shrub density favored dominant and opportunistic ant species, whereas later stages with increased tree and grass cover supported more diverse ant communities. These results demonstrate that fire drives successional specialization in ant communities through niche filtering. Maintaining heterogeneous fire‐age vegetation mosaics is therefore critical for conserving landscape‐level biodiversity in fire‐regulated mallee ecosystems.

## Graphical abstract

We sampled ants from five sites with varying fire histories (3, 5, 9, 26, and 34 years since the last fire). A total of 16,360 ants were collected, and 59 species across 20 genera were identified. Our results indicate that total ant abundance was higher in recently burnt sites and decreased with increasing time‐since‐fire, while ant species richness was higher in older burn sites. We also found that ant composition differed significantly in sites with different times‐since‐fire. Early post‐fire stages, characterized by high shrub cover, supported dominant and opportunistic ant species, whereas later successional stages, with greater tree and grass cover, were associated with higher ant species diversity.

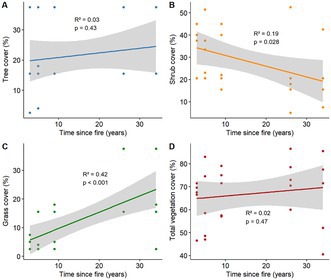

## 1 Introduction

Fire is a major disturbance in many regions worldwide (Doerr and Santín 2016; Meddens et al. 2018). It is increasingly recognized as a recurring process that regulates organismal traits, population sizes, species interactions, community composition, carbon and nutrient cycling, and overall ecosystem function (McLauchlan et al. 2020). Through these effects, fire supports global biodiversity (Kelly et al. 2020). Although studies have traditionally focused on plants (Vidal‐Cordero et al. 2023), growing attention is being directed towards understanding fire's impact on animals, including arthropods. Fire regimes and fire intensities are changing in many parts of the world due to increased global warming and a drying climate (Pechony and Shindell 2010); therefore, understanding how plants and animals respond to altered fire regimes is a primary concern for land managers and conservation biologists.

Ants are a dominant faunal group that respond to disturbance, especially fire, in keyways related to habitat openness and functional composition. Ants serve as an effective bioindicator species (de Lobry Bruyn 1999; Majer 1983) in ecosystem regeneration through changing richness and composition at different stages in restoration (Andersen and Sparling 1997; Lawes et al. 2017). Ants are important in maintaining biodiversity. Through mutualistic interactions such as seed dispersal (Del Toro and Ribbons 2019) and by protecting plants from herbivorous insects (Aranda‐Rickert et al. 2014), they can shape the plant communities. On the other hand, vegetation regeneration following disturbance influences the abundances and richness of ant communities (Fox et al. 2003).

Fire can affect ant communities and their diversity in multiple ways, including by directly killing individuals and indirectly through changing the vegetation structure (Staff et al. 2023). According to the habitat accommodation model (Fox et al. 2003), vegetation regrowth after fire shapes fauna distribution and abundance by altering the availability of suitable habitats (Gosper et al. 2019). Therefore, fire generally results in significant differences in the abundance and diversity of ants sampled at different times post‐fire. Burned habitats support a mix of species, including those that withstand the fire and adapt to the altered environment, as well as those that disperse from unburned areas, drawn by newly available resources (Vidal‐Cordero et al. 2023). For ants, the taxonomic responses to fire have been quite variable across the world. In the Mediterranean region, ant communities are generally higher in overall richness in burnt habitats (Vidal‐Cordero et al. 2023), while a similar study assessing time‐since‐fire effects on ant diversity in Australian mallee landscapes reported no clear relationship between time‐since‐fire and ant diversity (Staff et al. 2023), and However, given the higher diversity of ant genera in arid and semi‐arid regions (Andersen 2016; Hammer et al. 2015), it remains unclear how ant communities change after wildfires, both short‐term and long‐term, and the indirect influence of altered vegetation structure on these communities.

Australia has large arid and semi‐arid zones covering approximately 70% of the continent (Pudmenzky et al. 2015), and in Australian semi‐arid landscapes wildfires are frequent (Gosper et al. 2015). The state of New South Wales, where our research was conducted, and most of eastern Australia have been prone to frequent wildfires since at least the Middle Eocene (~40 million years ago; Nguyen et al. 2021). Given the ecological importance of this fire‐prone region, land management has been recognized as a conservation priority. In response, organizations such as the NSW Biodiversity Conservation Trust promote private land conservation by helping landowners manage their land to protect biodiversity. This includes restoring native vegetation that supports threatened species and ecosystems, reducing land clearing, and managing grazing pressure.

In this study, we examined how ant species richness, abundance, and composition varied across post‐fire successional stages in a semi‐arid mallee ecosystem in New South Wales, Australia. We made the following predictions:

1. Based on the habitat accommodation model (Fox 1982), we predicted that overall ant abundance would be higher in the early post‐fire stages, as simplified habitats tend to favor disturbance‐tolerant species (Andersen 2019), which can be due to increased availability of open space, resources, and reduced competition. Further, we predict that ant species richness will increase with time‐since‐fire due to modifications in habitat structure over time.
2. We predict that ant community composition will differ among sites with varying time‐since‐fire histories, with community dissimilarity increasing as the difference in time‐since‐fire between sites increases.
3. We predict that total vegetation cover will increase with time since fire, contributing to greater habitat complexity over time. However, shrub cover is expected to increase in the short term following fire (Van Etten et al. 2021), which may help explain observed changes in ant composition. These predictions are consistent with life‐form succession theory (Budowski 1965), which proposes that dominant plant forms (e.g., grasses, shrubs, and trees) change predictably through time following landscape disturbances.

## 2 Method

### 2.1 Study Site

This research was conducted at Nanya Research Station (33.1281° S, 141.3817° E) in the southwest of New South Wales, Australia. Nanya Station is a 40,000 ha conservation property supporting natural salt lakes, old‐growth *Eucalyptus* Mallee and a variety of intact ecosystems in the Scotia Mallee. Nanya Station is owned and managed by Federation University since 2004 and is surrounded by the Scotia wildlife Sanctuary and three privately owned properties.

The Scotia Mallee is considered cool semi‐arid with high temperatures in summer, with average maximum–minimum range of 32°C–16°C in February and mild temperatures in winter, with average minimum‐maximum range of 5°C–15°C in July. The mean annual rainfall is approximately 220 mm with an unpredictable rainfall pattern. Vegetation mainly consists of mallee ( *Eucalyptus gracilis* , *E. dumosa* , *E. socialis* ), open shrublands and open woodlands (*Casuarina pauper*, *Alectryon oleifolius*; Westbrooke 2016). The study sites where pitfall traps were placed had consistent vegetation, dominated by mallee eucalyptus and Triodia spinifex grass understorey.

**FIGURE A1:**
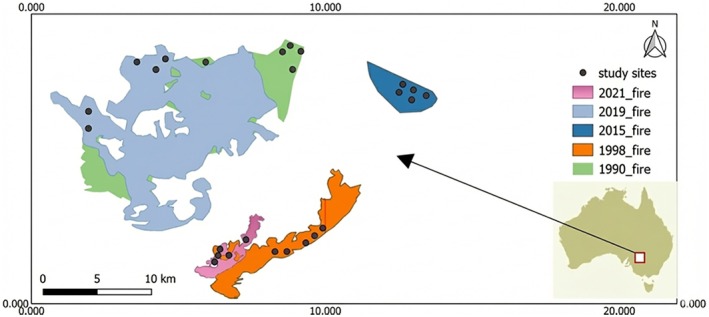
Location map of the study area.

We collected data from five areas (Appendix Figure A1) that differed in time‐since‐last‐fire (3, 5, 9, 26, and 34 years since fire, respectively). We established five transects within each area which were approximately 0.5 km apart and the data collections were conducted during summer 2024. Sites were selected by examining fire history GIS layers and confirmed the accessibility during a site visit in August 2023.

### 2.2 Vegetation Cover and Other Environmental Variables

**FIGURE A2:**
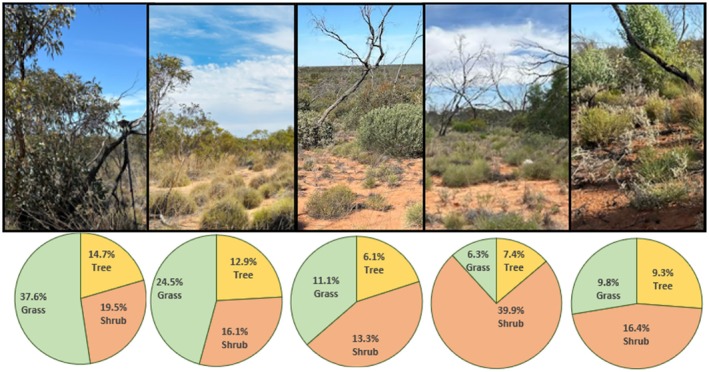
Photographs and corresponding pie charts depicting percent cover of grass, shrubs, and trees of vegetation in five fire ages (left to right: (a) 1990 (34 years), (b) 1998 (26 years), (c) 2015 (9 years), (d) 2019 (5 years), and (e) 2021 (3 years)).

To measure the vegetation cover we used the same transects where the pitfall traps were deployed. At 4‐m intervals along 20‐m transect, we recorded the number of plants and categorized them into lifeforms (grass, shrubs, and trees) to get cover estimates for each lifeform. Vegetation cover was estimated using the Daubenmire method, where we assigned the visual cover estimates to predefined percentage cover classes and then converted them to mid‐point value for analysis. Photographs were taken by a single person at each site to support cover estimates (Appendix Figure A2). We recorded the latitude, longitude, and elevation of sites using a handheld GPS.

### 2.3 Ant Sampling

Invertebrate sampling was conducted in February 2024. We used pitfall traps which are considered a straightforward and cost‐effective sampling method for ants. We used 250 mL open small jars filled with 150 mL of 80% propylene glycol solution. We placed five pitfall jars per 20‐m long transect, maintaining a 4‐m gap between the pitfall jars. For each site, we placed pitfall traps along five transects resulting in 25 total pitfall traps per site. The pitfall traps were left in the field for 48 h. Any biological material captured in pitfall traps was stored in 70% ethanol. Ants were separated from other invertebrates and identified and counted using a Nikon SMZ 747 dissecting microscope. We used the Australian ant identification book by Shattuck (1999) and AntWiki online database (AntWiki, n.d.) to help with the ant identification, and confirmed species and morphospecies identifications with the help of an expert (Greg Horrocks, Federation University Australia) familiar with the Nanya ant community.

### 2.4 Data Analysis

#### 2.4.1 Effect of Fire History on Ant Richness and Abundance

We first broadly characterized the ant community at Nanya Station by pooling all pitfall trap captures to construct rank‐richness and rank‐abundance curves that identified the richest and most abundant genera in the region.

Second, we quantified the effect of time‐since‐fire on different measures of the ant community. Ant abundance was taken as the total number of ants per transect (Number of transects = 25) and species richness was the total number of different species found per transect. We modeled ant abundance and species richness as functions of time since fire using generalized linear mixed models (GLMMs) fitted in R with the glmmTMB package (McGillycuddy et al. 2025). Time‐since‐fire was treated as an ordered factor to represent age classes, and orthogonal polynomial contrasts were used to assess non‐linear trends. For abundance, we used a negative binomial distribution with NB2 parameterization and a log link to account for overdispersion in count data. For species richness, we used a COM‐Poisson distribution with a log link to accommodate flexible dispersion (Sellers and Shmueli 2010). We included random intercepts for site and transect nested within site to acknowledge the lack of independence among transects within the same site and among pitfalls within transects. Model fit was assessed using AIC and residual diagnostics using the DHARMa package (Hartig 2024). Time‐since‐fire effects were evaluated using likelihood ratio tests comparing models with and without Time‐since‐fire, and effect sizes were summarized using estimated marginal means with 95% confidence intervals using the emmeans package (Lenth 2023). For visualization, we plotted estimated marginal means for time‐since‐fire classes alongside smooth predictions from spline‐based models treating time‐since‐fire as continuous.

#### 2.4.2 Fire History and Ant Community Composition

To determine how ant composition is structured through time‐since‐fire at our study sites, we conducted a permutation‐based multivariate analysis of variance (PERMANOVA), using the *adonis* function in the vegan package (Oksanen et al. 2008) based on Bray–Curtis dissimilarities of transformed presence/absence data for all species treating time‐since‐fire as a continuous (3–34), and their interaction as fixed effects. We used site ID as a random factor to account for the fact that different transects were sampled in one fire site. In addition to the overall model, we performed pairwise PERMANOVA comparisons between all fire‐age categories to identify specific differences in community composition among burn years. To visualize the separation between ant communities among sites sampled at different time‐since‐fires, we used PCA. For the 12 most abundant ant species, frequency of occurrence per transect was calculated as the proportion of pitfalls containing individuals. Species‐specific GLMMs were fitted using the lmer function, with frequency as the response, time‐since‐fire as an ordered factor fixed effect and transect as a random effect. Estimated marginal means and 95% confidence intervals were extracted using emmeans. For visualization, we plotted estimated marginal means for time‐since‐fire classes from the abundance, richness and ant species models alongside smooth predictions from spline‐based models treating time‐since‐fire as continuous.

#### 2.4.3 Fire History and Vegetation Cover

**FIGURE A3:**
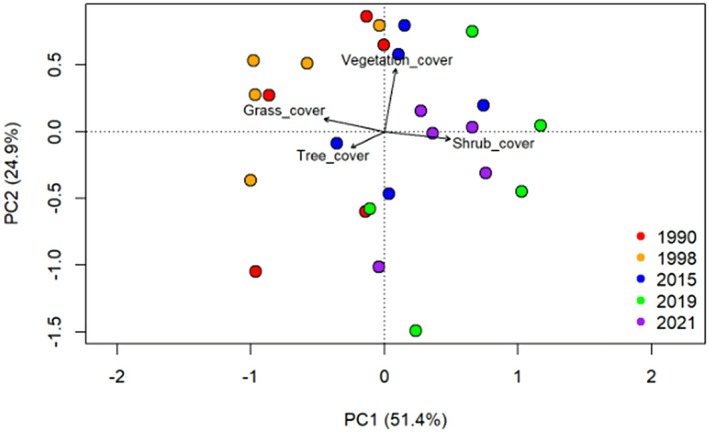
PCA ordination plot showing the distribution of study sites and environmental vectors.

We used the g*lmer* function in the library lme4 of R Core Team 4.2.0 to explore the effect of time‐since‐fire (TSF) on vegetation components, including shrub cover, tree cover, grass cover, and total vegetation cover, and by taking transect as a random factor. As these response variables (e.g., shrub cover) were recorded as counts, we specified a Poisson error distribution with a log link function. Further we used PCA to extract PC1 variables of vegetation components (Appendix Figure A3) to evaluate the effect of vegetation components on ant species richness (Appendix Figure A2). Then, extracted PC1 scores were used in the generalized linear models by accounting for transect as a random factor.

## 3 Results

### 3.1 Overview of the Ant Community

**FIGURE 1:**
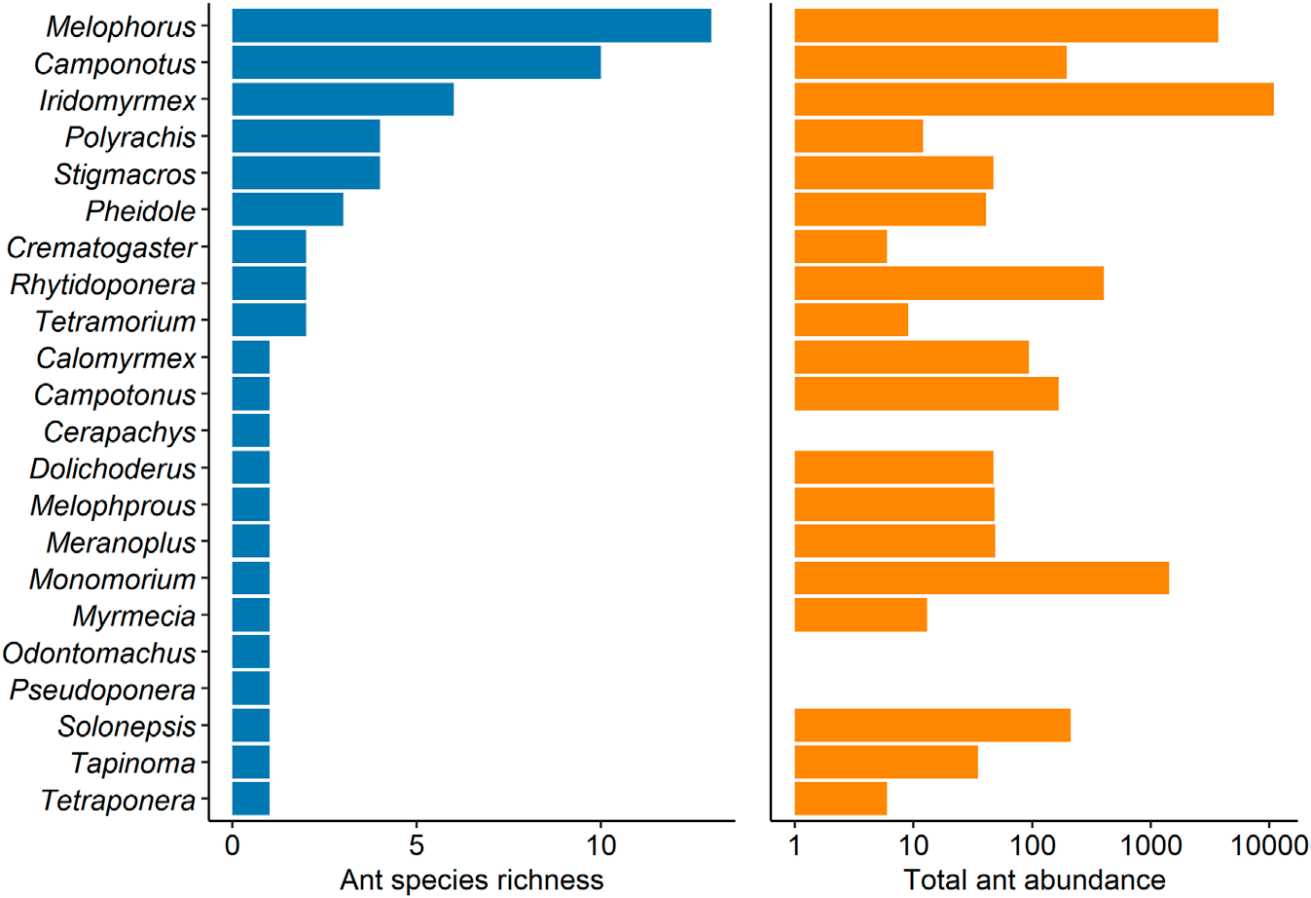
Richness and abundance within genera in ant communities at Nanya Research Station.

We identified 59 species from 20 genera (Appendix Table A1) from a total of 16,360 ants collected from our pitfall traps at Nanya Research Station. *Melophorus* (14 species) was the best represented genus, followed by *Camponotus* with 11 species and *Iridomyrmex* with six species (Figure 1). *Iridomyrmex* was the most abundant genus, followed by *Melophorus* and *Monomorium*.

### 3.2 Fire History and Ant Richness and Abundance

**FIGURE 2:**
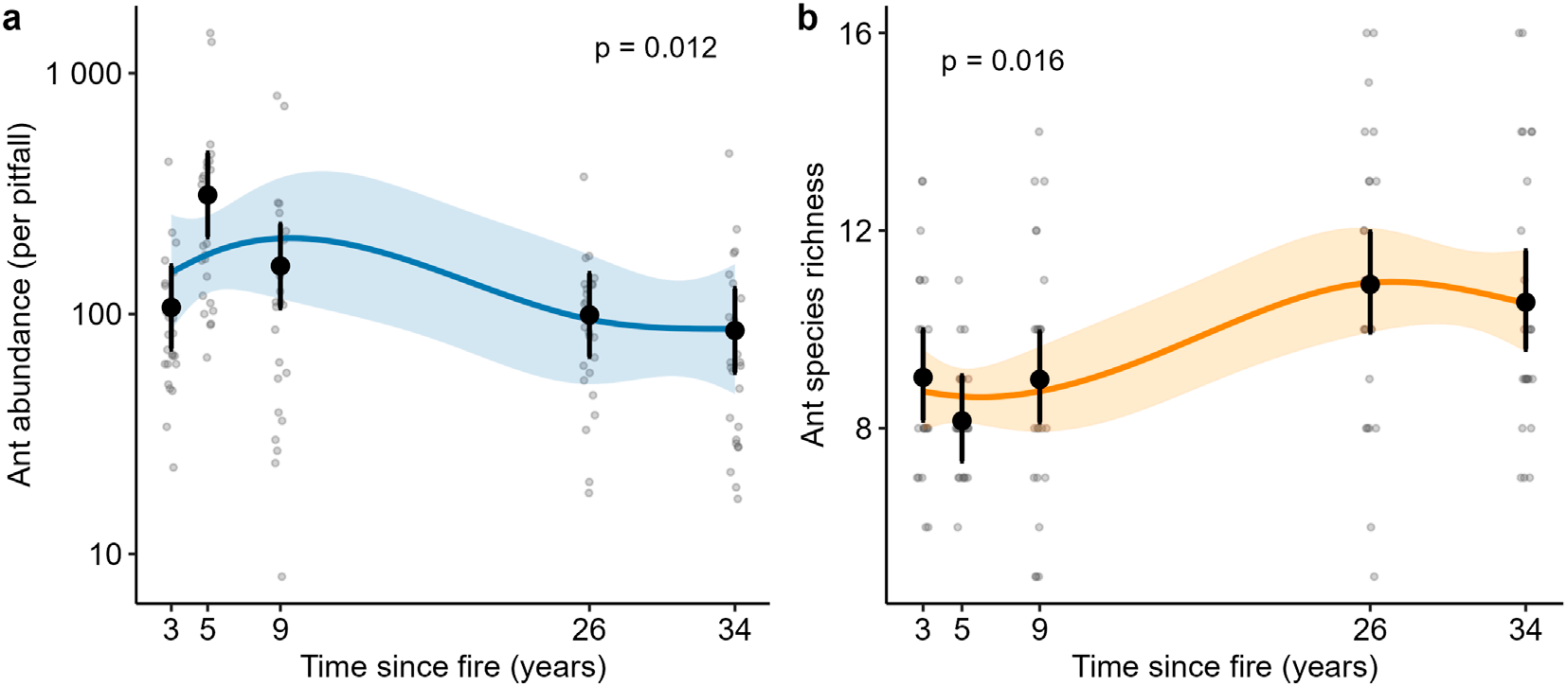
Model‐predicted ant abundance (a) and species richness (b) across time since fire (TSF). Each panel shows raw pitfall data (gray jittered points), estimated marginal means (black points with 95% confidence intervals) from categorical Time‐since‐fire models, and smooth predictions (line with 95% CI ribbon) from spline‐based models treating Time‐since‐fire as continuous. Abundance was modeled using a negative binomial GLMM (NB2) with a log link, and richness using a COM‐Poisson GLMM to accommodate flexible dispersion. The *p* value presented show the time‐since‐fire effect from likelihood ratio tests comparing models with and without time‐since‐fire.

Ant abundance declined with time since fire (Figure 2), with a significant overall time‐since‐fire effect (likelihood ratio test *χ* ^2^_4_ = 12.8, *p* = 0.012), and the orthogonal polynomial contrasts showed clear non‐linearity (linear *p* = 0.0135; quadratic *p* = 0.0034; cubic *p* = 0.0013), under moderate overdispersion (NB2 *θ* = 1.98). Ant species richness increased with time since fire (*χ* ^2^_4_ = 12.1, *p* = 0.016), driven by a positive linear component (*p* = 0.00014) with mild non‐linearity (cubic *p* = 0.0077), consistent with overdispersion relative to Poisson (COM‐Poisson ν = 0.509).

### 3.3 Fire History, Ant Community Composition, and Vegetation Composition

Results obtained from the PERMANOVA revealed a significant overall effect of time‐since‐fire on ant community composition (df = 4, *F* = 4737.8, *R* ^2^ = 0.999, *p* = 0.001) and pairwise comparisons indicated that ant communities among post fire‐year categories were significantly different from each other (*p* < 0.05). However, the magnitude of differences varied: the ant communities of recently burned sites (2021 (3 years) time‐since‐fire) were most distinct, especially compared to older fire sites (e.g., 1990 (34 years) time‐since‐fire vs. 2021 (3 years) time‐since‐fire, *F* = 12,277, *p* = 0.007). In contrast, older burns such as 1990 (34 years) and 1998 (26 years), although statistically different (*F* = 828.95, *p* = 0.009), showed lower *F* value, indicating an increased similarity in ant community composition.

**FIGURE 3:**
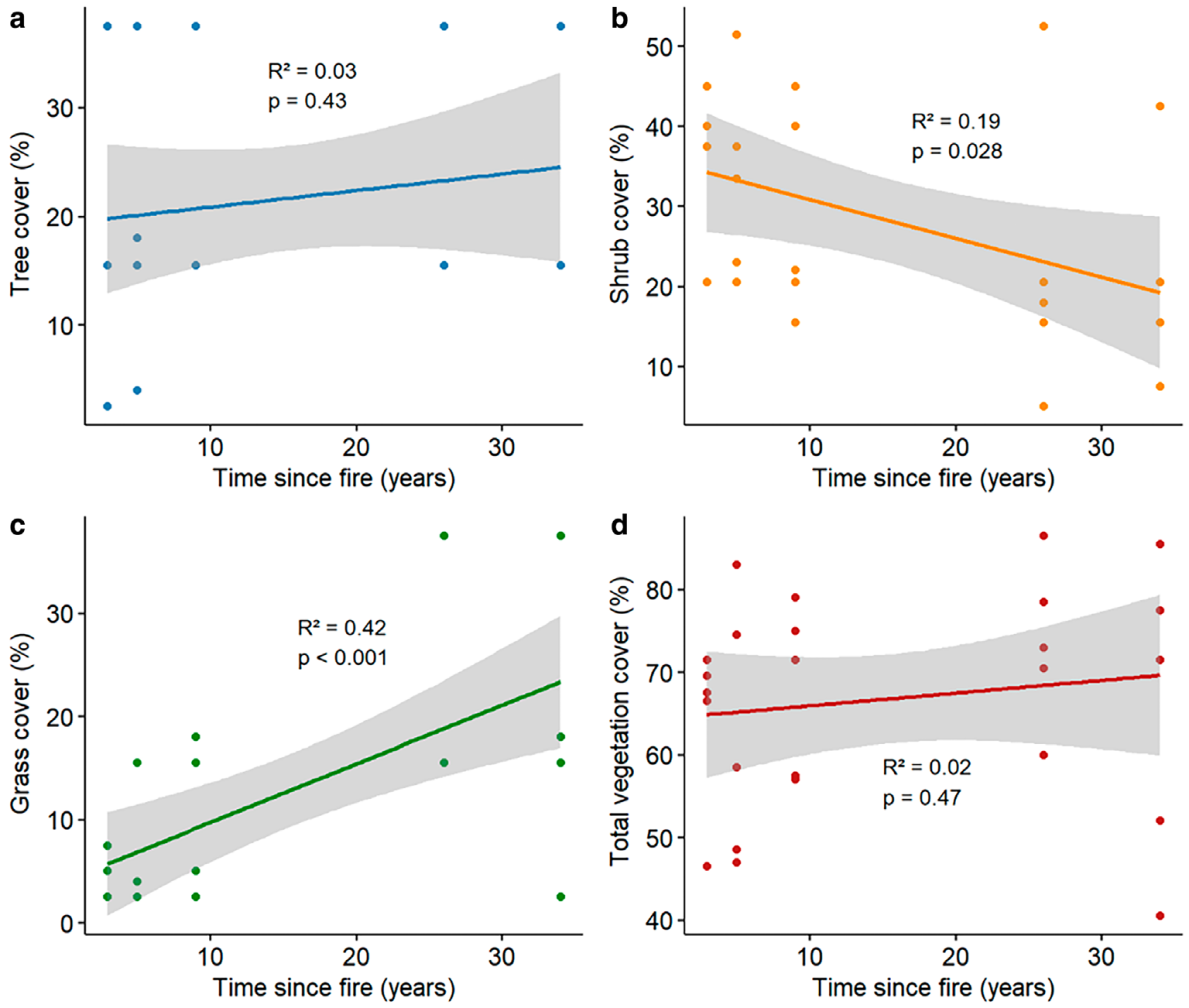
(a) Effect of time‐since‐fire on tree cover, (b) effect of time‐since‐fire on shrub cover, (c) effect of time‐since‐fire on grass cover, and (d) effect of time‐since‐fire on total vegetation cover. The shaded area indicates the 95% confidence interval.

Both grass cover and tree cover showed a positive correlation with increasing time‐since‐fire, whereas shrub cover showed a negative correlation and found that the vegetation variables were correlated (Figure 3). The first principal component (PC1) explained 51.4% of the total variance in vegetation structure and represented a gradient from grass‐ and tree‐dominated sites (negative loadings) to shrub‐dominated sites (positive loadings). The variable loadings on PC1 were shrub cover (1.53), grass cover (−1.41), tree cover (−0.79), and total vegetation cover (0.26), indicating that variation among sites was primarily explained by shrub cover (Appendix Figure A3).

**FIGURE 4:**
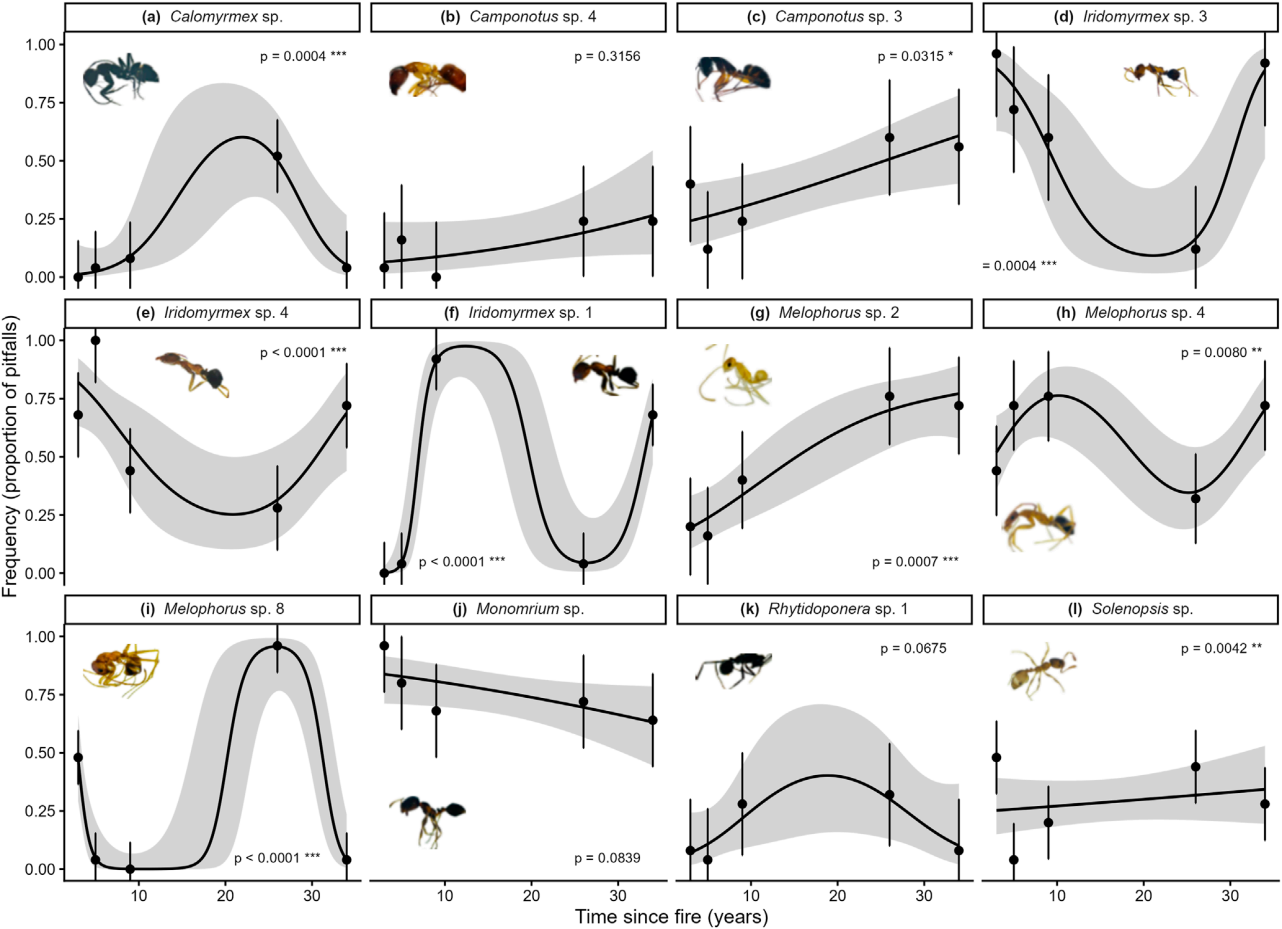
Predicted frequency of ant species across time since fire. Points represent estimated marginal means (EMMs) from species‐specific generalized linear mixed models (frequency ~ time‐since‐fire, with transect as a random effect), with 95% confidence intervals shown as error bars. Shaded ribbons and lines represent continuous species‐specific predictions from generalized additive models fitted to the same data. The *p* values indicate the significance of the effect of time since fire from the GLMMs.

Individual ant species showed a range of responses to time‐since‐fire (Figure 4). Key species that showed an increase in occurrence with time‐since‐fire were *Camponotus* sp.4 and sp3 (Figure 4b,c) and *Melophorus* sp3 (Figure 4g). Notably, there were no ants that showed a decrease in occurrence over time‐since‐fire.

## 4 Discussion

**FIGURE 5:**
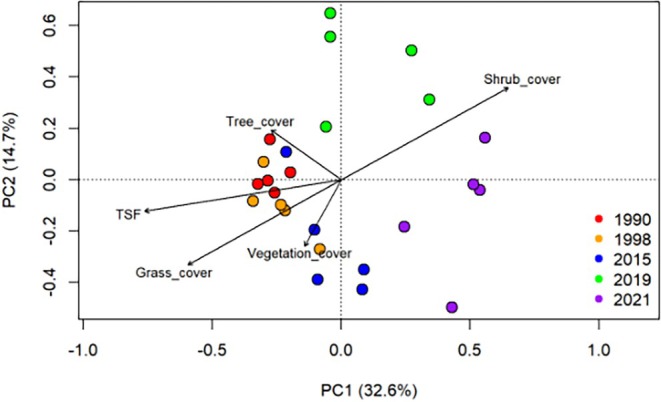
Ordination plot of principal component analysis (PCA) showing the differences in ant species composition from sites grouped by time‐since‐fire. Different colored dots represent individual transects within the different fire patches (1990 (34 years), 1998 (26 years), 2015 (9 years), 2019 (5 years), and 2021 (3 years)) where ants were collected. Arrow vectors indicate the direction and strength of correlations between environmental variables and ant species composition.

In this study, we explored how ant communities and vegetation structure change in response to the time‐since‐fire in a semi‐arid mallee landscape. Ant species richness showed a weak positive correlation with time‐since‐fire while total ant abundance showed a negative trend (Figure 2). We also found that the ant community composition was different among different post‐fire stages and the dissimilarity in ant composition was higher in recently burnt sites (3–5 years of time‐since‐fire) (Figure 5). Our results indicate that time‐since‐fire modifies habitat structure by influencing shrub, tree, grass, and total vegetation cover, which in turn may affects ant community composition at the landscape scale. Below we discuss our findings and reflect on the implications for biodiversity conservation in semi‐arid ecosystems.

One of our key findings was that overall ant abundance was higher soon after fire than in the long unburnt sites. In contrast, the species richness showed positive correlation to the increasing time‐since‐fire resulting higher species diversity over the 25–35 timeframe. The inverse relationship between ant abundance and species richness across the post‐fire gradient is consistent with the habitat succession model (Fox 1982; Staff et al. 2023) where increasing habitat complexity over time provides a wider range of resources and niches that support higher ant species richness. Time‐since‐fire significantly influenced ant abundance and richness, and the ant taxonomic variations were better explained by observed changes in the vegetation structure along the post‐fire successional gradient (Appendix Figure A2). Habitat type is an important predictor of ant community responses to fire (Vasconcelos et al. 2017), and similar studies in semi‐arid habitats (Andersen 1983; Staff et al. 2023; Vasconcelos et al. 2017), found wildfires have an indirect influence on ant diversity through modification of the vegetation structure (Andersen et al. 2014; Brassard et al. 2023; Knudsen et al. 2024). Immediately after a fire, the simplified vegetation structure results in open habitats (Parr et al. 2007), favoring the dominant *Iridomyrmex* (Andersen 2019), hot climate specialists such as *Melophorus*, and opportunistic species such as *Rhyitodoponera* (Hoffmann 2003), all capable of exploiting available resources may lead to higher total ant abundance. As vegetation recovers and habitat complexity increases, it provides a wider array of niches, facilitating the colonization and coexistence of more diverse ant species (Calle et al. 2013; Nooten et al. 2019). However, the total abundance may decrease as competition between species increases with resources shared among more species.

**FIGURE A4:**
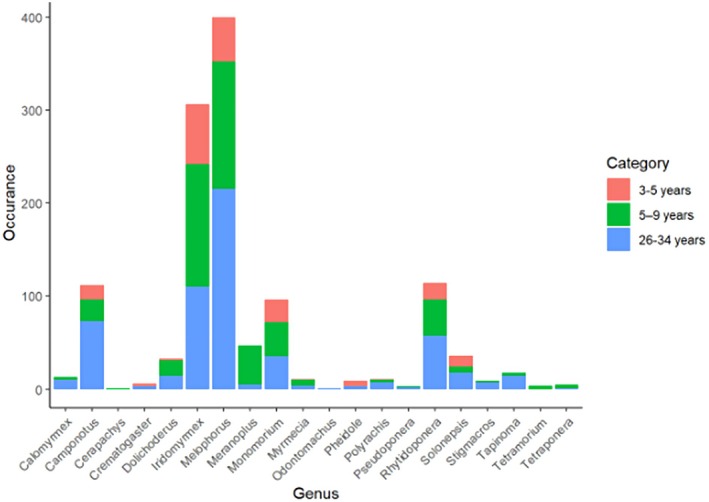
Variation in ant genus occurrence along a post‐fire age gradient (0–3, 5–9, and 26–34 years since fire).

Our results also showed that ant community composition varied significantly across sites in different successional stages (Figure 5), confirming our second prediction. We found that recently burned sites (3–5 time‐since‐fire) were compositionally distinct from other sites and had greater variability in ant assemblage composition, whereas long burned sites converged and shared similar species. Therefore, based on our results, it shows 25–35 years is an approximate time frame for ant communities to increase in species richness in this fire prone mallee landscape. We observed genera such as *Rhytidoponera*, dominant *Iridomyrmex*, and arid‐adapted *Melophorus* in all burnt sites (Appendix Figure A4), and this pattern has been observed in many other Australian dry eucalypt regions. Andersen (2007) reported that most arid regions are particularly dominated by dolichoderines and rich in *Melophorus* diversity. The high abundances of dolichoderines can be explained by favorable vegetation structure, high temperatures, and access to resources (Bestelmeyer 2000). The number of individuals in dominant dolichoderine (*Iridomyrmex*) and specialist predators such as *Stigmacros*, *Myrmecia*, and *Cerapachys* was higher in recently burnt habitats. Habitat complexity strongly affects the structure and dynamics of ecological communities, with increased complexity often leading to greater species diversity (Barton et al. 2024) through promoting species coexistence by providing a wide range of niches, thereby reducing niche overlap and increasing species richness. Similar patterns have been recorded elsewhere; the bare ground exposed by fire has provided an advantage for thermophilic ant species, *Iridomyrmex, Melophorus* in Australia (Andersen 2019; Hoffmann 2003), *Forelius pruinosus* in pine savannas in Florida (Izhaki et al. 2003), and *Aphaenogaster senilis* in Mediterranean forests (Vidal‐Cordero et al. 2023). Ants belonging to the Subordinate Camponotini (Andersen 2019) were observed at all sites, coexisting with dominant Dolichoderinae ants. Their abundance was higher in older burnt habitats (26, 34 years of time‐since‐last fire). Same behaviors were shown by Mediterranean ant communities; Vidal‐Cordero et al. (2023) reported that *Camponotus lateralis* was more likely to occur in areas that had not burned for at least 15 years, where vegetation had re‐established.

For our third prediction, we examined the effect of time‐since‐fire on vegetation structure, and how it may influence the ant composition within the landscape. We found that the shrub cover (*p* = 0.001) and grass cover (*p* = 0.002) mainly explained the variance observed in ant composition among different post‐fire stages (Figure 5, Table 1). For instance, we observed higher ant richness in older burnt habitats, where the restored vegetation (Appendix Figure A2) has created favorable conditions for various ant species, including litter‐dwelling genera such as *Crematogaster*, *Tetramorium*, and *Pheidole*. The results of the GLMM showed a weak and non‐significant relationship with ant species richness (AIC = 104, marginal *R* ^2^ = 0.035), although the availability of shrubs and grasses may influence microclimate and thereby shape ant community composition within the landscape. The availability of a higher number of grasses such as *Triodia scarisosa*, *Austrasipa nullanulla*, *Lomandra leucocephala*, and *Eragrostis eriopoda* and the presence of regrowing different species of mallee trees in older burnt sites shaped the microclimatic conditions (Achury et al. 2022), and the accumulation of leaf litter provided suitable nesting habitats for litter‐dwelling ant species (Barton et al. 2024; Groc et al. 2017). Clarke et al. (2010) found that time‐since‐fire strongly influences the abundance and structure of plant communities with regeneration of mallee eucalypt vegetation in semi‐arid ecosystems and allows most animal species to display a discrete set of generalized responses to time‐since‐fire. Further, Brassard et al. (2023) and Andersen (2019) also suggested that woody cover is a strong predictor of variation in ant communities in Australian savannas and it was shaped by the long fire history of the region.

**TABLE 1:** Summary of results of principal component analysis.

Vectors	PC1	PC2	*R* ^2^	*p*
Time‐since‐fire (TSF)	−0.987	−0.159	0.621	0.001
Shrub cover	0.875	0.484	0.570	0.001
Grass cover	−0.873	−0.488	0.483	0.002
Tree cover	−0.815	0.579	0.116	0.234
Total Vegetation cover	−0.484	−0.875	0.090	0.375

In early successional stages, shrub abundance was particularly high, consistent with studies reporting shrub‐dominated succession patterns in similar ecosystems (Hodgkinson 1998; Kenny et al. 2018; Vasques et al. 2023). Shrubs such as *Acacia* spp. in the early successional stages may provide multiple benefits for ant communities, including food resources such as seeds and extrafloral nectar resources for behaviourally dominant ants. For instance, Oliveira et al. (2024) reported that fire increased the incidence of ant–plant interactions associated with extrafloral nectar on *Acacia*, with dominant dolichoderine ants accounting for approximately 85% of all recorded interactions.

## 5 Implications and Conclusions

Changes in ant assemblages among post‐fire stages, together with species‐specific differences in habitat use, have important implications for land management and highlight the potential value of maintaining heterogeneous fire regimes. Our results indicate that sites with different time‐since‐fire support distinct ant assemblages, suggesting that a combination of recently burned and long‐unburned habitats may contribute to greater variation in ant community composition at the landscape scale. Although long‐unburned sites supported higher local species richness, different post‐fire stages may collectively enhance regional diversity in fire‐prone semi‐arid landscapes.

Time‐since‐fire effects on ant assemblages and habitat structure should be an important consideration in biodiversity conservation. Changes to fire regimes in semi‐arid regions therefore can be expected to have large effects on vegetation and ant community composition. The pyro diversity hypothesis suggests that spatial and temporal heterogeneity of fire regimes promotes biodiversity by creating a mosaic of habitats with varying successional stages (Bowman et al. 2016; Brassard et al. 2023; Tingley et al. 2016), and our results are more consistent with this, as sites with varying time‐since‐fire supported distinct ant assemblages, contributing to higher ant richness within the landscape. We found that fire acted as a niche‐filtering mechanism, with long‐term effects favoring many species and short‐term effects supporting the abundance of some species. However, morphological traits of ants may also help predict community assembly in different habitat structures and disturbance regimes, thereby improving the ability to categorize communities and assess the effects of global change.

## Author Contributions

**Norma L. Fernando:** conceptualization (lead), data curation (lead), formal analysis (lead), funding acquisition (supporting), investigation (equal), methodology (lead), project administration (equal), resources (equal), software (lead), supervision (equal), visualization (lead), writing – original draft (lead), writing – review and editing (equal). **Nick L. Schultz:** data curation (equal), formal analysis (equal), investigation (equal), software (equal), supervision (equal), writing – review and editing (equal). **Grant Palmer:** conceptualization (supporting), formal analysis (supporting), funding acquisition (equal), investigation (equal), project administration (equal), supervision (equal), writing – review and editing (supporting). **Philip S. Barton:** conceptualization (equal), data curation (supporting), formal analysis (equal), funding acquisition (equal), investigation (equal), methodology (supporting), project administration (supporting), resources (supporting), software (supporting), supervision (lead), validation (equal), visualization (supporting), writing – review and editing (equal).

## Funding

This research was supported by Federation University Australia (30439463) and NSW Biodiversity Conservation Trust (G2467).

## Conflicts of Interest

The authors declare no conflicts of interest.

## Data Availability

The data supporting the findings of this study are available in Figshare at https://doi.org/10.25955/31081192.

## Appendix A

**TABLE A1:** Summary of ants collected from Nanya Station, NSW.

Sub family	Species code	Morphospeicies	Count
Dolichoderinae	msp3	*Dolichoderus*	167
Dolichoderinae	msp9	*Iriodomyrmex 1*	3041
Dolichoderinae	msp11	*Iriodomyrmex 2*	607
Dolichoderinae	msp15	*Iriodomyrmex 3*	634
Dolichoderinae	msp18	*Iridomyrmex 4*	5590
Dolichoderinae	msp37	*Iridomyrmex 5*	62
Dolichoderinae	msp56	*Iridoymyrmex 6*	20
Dorylinae	msp57	*Cerapachys*	1
Ectatomminae	msp2	*Rhytidoponera1*	310
Ectatomminae	msp24	*Rhytidoponera 2*	115
Formicinae	msp1	*Camponotus1 (Camponotus loweryi)*	47
Formicinae	msp4	*Camponotus 2*	117
Formicinae	msp5	*Camponotus 3*	4
Formicinae	msp6	*Melophorus 1*	306
Formicinae	msp7	*Melophorus 2*	2
Formicinae	msp8	*Melophorus 3*	351
Formicinae	msp10	*Melophorus 4*	937
Formicinae	msp12	*Melophorus 5*	317
Formicinae	msp19	*Polyrachis 1*	9
Formicinae	msp21	*Camponotus 4*	14
Formicinae	msp23	*Melophorus 6*	88
Formicinae	msp25	*Calomyrmex*	94
Formicinae	msp26	*Melophorus 7*	66
Formicinae	msp27	*Melophorus 8*	1374
Formicinae	msp28	*Melophorus 9*	33
Formicinae	msp32	*Melophorus 10*	20
Formicinae	msp33	*Camponotus 5*	9
Formicinae	msp35	*Campotonus 6*	6
Formicinae	msp36	*Polyrachis 2*	1
Formicinae	msp38	*Melophorus 11*	2
Formicinae	msp39	*Stigmacros 1*	2
Formicinae	msp40	*Stigmacros 2*	20
Formicinae	msp41	*Camponotus 7*	14
Formicinae	msp42	*Melophorus 12*	92
Formicinae	msp44	*Stigmacros 3*	24
Formicinae	msp47	*Camponotus 8*	7
Formicinae	msp48	*Camponotus 9*	6
Formicinae	msp49	*Stigmacros 4*	1
Formicinae	msp50	*Melophorus 13*	34
Formicinae	msp51	*Polyrachis 3*	1
Formicinae	msp52	*Camponotus 10*	2
Formicinae	msp53	*Melophorus 14*	48
Formicinae	msp54	*Camponotus 11*	1
Formicinae	msp58	*Polyrachis 5*	1
Myrmcinae	msp59	*Crematogaster 2*	2
Myrmiciinae	msp45	*Myrmecia*	13
Myrmicinae	msp13	*Crematogaster 1*	4
Myrmicinae	msp14	*Monomorium 1*	1427
Myrmicinae	msp16	*Tetraponera*	6
Myrmicinae	msp17	*Tapinoma*	52
Myrmicinae	msp20	*Solenopsis*	157
Myrmicinae	msp22	*Tetramorium 1*	7
Myrmicinae	msp30	*Meranoplus*	49
Myrmicinae	msp31	*Odontomachus*	1
Myrmicinae	msp34	*Pheidole 1*	32
Myrmicinae	msp43	*Pheidole 2*	3
Myrmicinae	msp46	*Pheidole 3*	6
Myrmicinae	msp55	*Tetramorium 2*	2
Ponerinae	msp29	*Pseudoponera*	2
